# A Genetic-Algorithm-Based Optimization Routing for FANETs

**DOI:** 10.3389/fnbot.2021.697624

**Published:** 2021-06-15

**Authors:** Xing Wei, Hua Yang, Wentao Huang

**Affiliations:** ^1^School of Computer Science and Information Technology, Guangxi Normal University, Guilin, China; ^2^School of Computer Science and Engineering, Guilin University of Aerospace Technology, Guilin, China; ^3^School of Computer Science and Information Technology, Guangxi Normal University, Guilin, China

**Keywords:** routing protocol, artificial intelligence, flying *ad hoc* network, genetic algorithm, routing optimization

## Abstract

In view of the characteristics of high mobility of FANETs nodes, combined with the features of Topology-based class routing protocol on-demand search, a Genetic-algorithm-based routing (GAR) protocol is proposed for FANETs which based on improved genetic algorithm for FANETs route search, and it taking into account the link stability, link bandwidth, node energy, and other factors. GAR improves the selection, crossover, and variation operators of the genetic algorithm so that GAR can finally plan an optimized route from the communication initiating node to the destination node quickly using a smaller cost. The experimental results show that GAR can largely improve the throughput, reduce the delay and improve the stability of the network, which is more suitable for FANETs.

## Introduction

Flying Ad Hoc Network (FANET), as a new type of mobile *ad hoc* network (Chriki et al., 2019; Shakhatreh et al., 2019; Khan et al., 2020; Nawaz et al., 2020), uses aircraft as a converter for transmitting, receiving or forwarding wireless communication in the air. It can be replaced at any time without the assistance of any fixed facilities. At any time, set up a network at any place to achieve high-efficiency communication at the network layer of the multi-aircraft system. With the advancement of embedded systems and the miniaturization of electromechanical systems, micro-UAVs can monitor in the air, detect biological agents, identify targets, and relay communications due to their low cost, strong scalability, easy concealment, and difficulty in tracking. It has a wide range of application scenarios (Chriki et al., 2019; Mozaffari et al., 2019; Wei and Yang, 2020a), and the flying *ad hoc* network with micro-UAV as the radar has attracted a lot of attention from the academic, industrial, and military circles. Compared with the traditional wireless network index, the flight *ad hoc* network mainly completes the communication process through data transmission between each other. Routing protocols are the key technology to enhance network performance and to ensure proper network communication.

A FANET can be expressed as an undirected graph G = (V, E), V represents the set composed of all nodes vi, and E represents the set composed of all links ei. The potential of V remains unchanged, but the potential of E changes with the establishment and deletion of links, and the optimal route is to find a suitable set {v1, v2,…, vn} under certain conditions, so that the corresponding the set {e1, e2, …, en} is the one that with the least cost and the highest stability. For FANETs with n nodes, there are n! total permutations of n nodes. In a wireless network such as FANETs, the route direction is usually not limited, so it is a harmonious combination. Under the conditions, the total number of routes will reach n!/2n. If the exhaustive search method is used, all the cases are taken into account, and all routes need to be found, and then they are compared separately to find the optimal route, thus finding the best route. The optimal route is too weighty for battery-powered FANETs to be realized at all. Therefore, the usual approach is to determine a usable route, not an optimized route.

In FANETs, when an intermediate node has an obligation to communicate, all neighbor nodes of the intermediate node can be regarded as the next hop routing node of the route. For FANETs, finding a stable, good-performance optimized route becomes an NP-hard (Yang and Liu, 2019; Wei and Yang, 2020a,b). As a kind of artificial intelligence algorithm, genetic algorithm, its powerful global search ability, especially its search ability to complex optimization problems, makes genetic algorithm (GA) widely used in the research of path planning, task allocation and other problems. The routing planning problem in FANETs can be regarded as a single-objective planning problem, so it is a feasible option to introduce heuristic algorithms such as genetic algorithm into FANETs routing planning.

## Related Works

### FANETs Routing Protocol

According to published literature, in order to cope with various complex scenarios in FANETs, researchers have designed various routing protocols, among which one of the more common ones is the topology-based routing protocol. Among the topology-based routing protocols, they are mainly divided into static routing, proactive routing, reactive routing, and hybrid routing.

Static routing is derived from traditional wired networks and is only applicable when the network topology does not change, but not for MANETs and FANETs where the network topology changes frequently. In network planning, routing tables need to be pre-designed and stored in each network node. At the same time, the routing table of static route cannot be updated as the network changes, so it can only be used with the communication at the terrestrial base station, and when the communication link fails, it will interfere with the communication of the whole network.

Proactive routing uses broadcast routing tables to maintain the routing tables of nodes, and all nodes in the network contain up-to-date routing information to other destination nodes. The advantage is that routes to the destination nodes are found immediately when the nodes need data transmission. The drawback is that all nodes need to maintain real-time structural information about the network topology, and when the network topology changes, a large amount of routing update information will flood the network, which will be a great drain on FANETs with limited node energy and bandwidth resources. Some of the more famous ones are OLSR (Jacquet et al., 2003), TDBRF (Ogier et al., 2004), DSDV (He, 2002) etc.

Reactive routing is also an “on-demand” routing, i.e., the route searching process is initiated when a node needs to communicate and there is no route to the destination node in the routing table. Typically, it includes route searching, route maintenance, and error handling. The advantage is that nodes do not need to maintain network-wide routing information and only need to initiate route maintenance or error handling when the topology changes, which can save a lot of bandwidth and node energy. The disadvantage is that the exchange of information during route finding brings high latency. Therefore, improving the convergence speed of the route finding algorithm of reactive routing and applying it to FANETs will achieve better results. The more famous algorithms in reactive routing are AODV (Perkins et al., 2003; Yang et al., 2017), DSR (Johnson et al., 2001; Yang et al., 2019), etc.

Hybrid routing protocols are a compromise that combines the advantages of proactive and reactive routing, with some nodes using proactive routing protocols and some nodes using reactive routing protocols. The efficiency of hybrid protocols depends on the number of other activated nodes, but the response to traffic demand depends on the number of traffic. Typical representatives are the Zone Routing Protocol (ZRP) algorithm (Haas and Pearlman, 2001), the Distributed Dynamic Routing (DDR) algorithm (Nikaein et al., 2000), and the algorithms that improve on them.

### Genetic Algorithm

**Figure 1:**
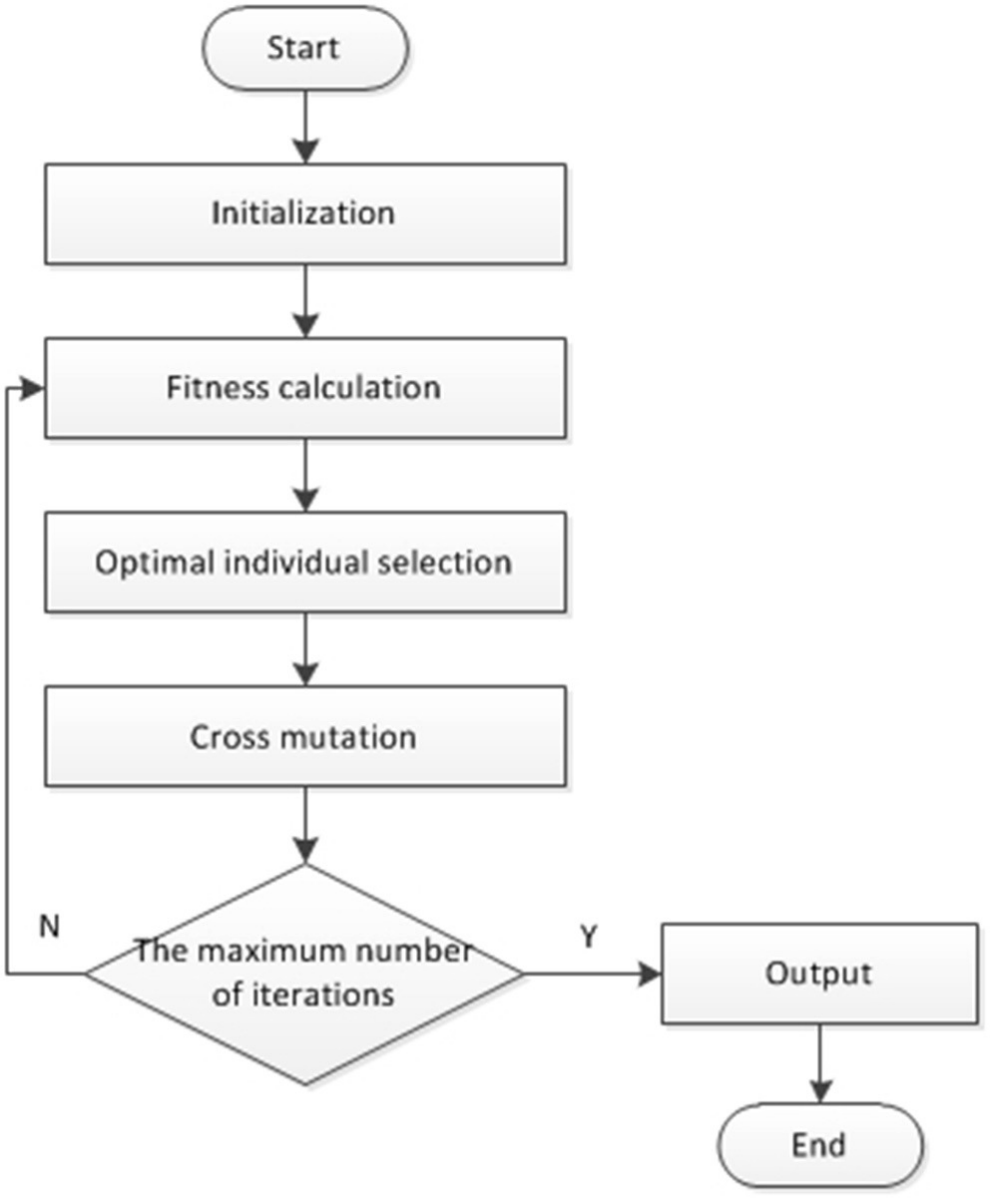
Main process of genetic algorithm.

Genetic algorithms, also known as evolutionary algorithms or genetic evolutionary algorithms (Holland, 1992; Weile and Michielssen, 1997; Lambora et al., 2019; Song et al., 2019), were first proposed by Professor Holland in the United States as a parallel and stochastic optimization search method that simulates the genetic laws and evolutionary mechanisms of a population of organisms, which can lead to the gradual evolution of the population to become better and better, and then obtain the optimal solution. The central theory of biological evolution is “survival of the fittest,” and genetic algorithms deepen it by emulating the unique nature of biological evolution to solve optimization problems. Genetic algorithms are probabilistic algorithms that search for the global optimal solution, abstracting the optimization problem into a parametric coding form, designing the fitness function with reference to the actual problem, calculating the fitness value of each individual in the population, and obtaining the next generation population through selection, crossover and mutation operations. In the process of genetic evolution, the individuals in the population will be retained with different probabilities according to their fitness values, and those with high fitness will be more likely to enter the offspring population, while those with low fitness may be eliminated, and the new population will have more advantages than the previous generation. The optimal solution to the optimization problem is output (Abualigah and Diabat, 2020; Selvaraj and Choi, 2020). The main process is shown in Figure 1.

## Optimization Method

### Objective Function

In FANETs, for an available route, the stability of the link, the bandwidth of the link, and the energy of the node are constrained to be considered comprehensively, and after optimization should have good stability, balanced bandwidth, and balanced energy of the node, etc., and can be summarized as the expression of the integrated cost function of routing between two nodes as in Equation (1).

(1) $$R_{ij}=\omega_{1}LS_{ij}+\omega_{2}\left(P_{i}+P_{j}\right)+\omega_{3}B_{ij}$$

where, ω is the weight adjustment parameter and has ω_1_ + ω_2_ + ω_3_ = 1. *LS*_*ij*_ is the link stability between node *i* and node *j*. Its calculation is referred to paper (Wei and Yang, 2020b) and the function expression is given in Equation (2). *P*_*i*_ is the residual power of the node *i*. *B*_*ij*_ is the link bandwidth between node *i* and node *j*.

(2) $$LS_{ij}=\frac{-\left(ab+cd\right)+\sqrt{\left(a^{2}+c^{2}\right)\cdot r^{2}-{\left(ad-bc\right)}^{2}}}{a^{2}+b^{2}}$$

The optimized route should have good stability, bandwidth balance, and node energy balance, so the objective function can be defined as:

(3) $$R_{l}=\sum_{\begin{array}{c} i\\ j\\ i\neq j \end{array}}\left(\omega_{1}LS_{ij}+\omega_{2}\left(P_{i}+P_{j}\right)+\omega_{3}B_{ij}\right)$$

### GA Coding Method

**Figure 2:**
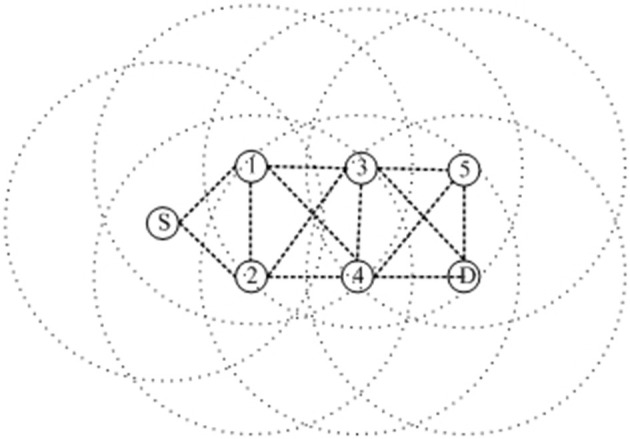
Network schematic with five intermediate nodes.

The encoding method directly affects the operation of genetic operators such as crossover operator and variation operator of genetic algorithm, so it largely determines the efficiency of genetic evolution. The common encoding methods are binary encoding, decimal encoding, etc. However, in FANETs, the number of intermediate nodes of the route to be optimized is not necessarily the same, so the encoding method needs to be improved to the traditional encoding method. A variable-length encoding method based on node *IDs* is designed here. i.e., node *IDs* represent genes in chromosomes, a chromosome is a route, the length of the chromosome is the same as the number of nodes in the route, and the length of the chromosome is how many nodes are in the route. Because the length of every route is not same, the length of the chromosome is also not consistent. The first generation chromosome represented by a FANET network containing 5 intermediate nodes as shown in Figure 2 can be represented as: *{S,1,2,3,D}, {S,1,2,3,4,D} {S,1,2,3,4,5,D}, {S,1,2,4,D}, {S,1,3,D}, {S,1,3,5,D}, {S,1,3,4,D}, {S,1,3,D}, {S,1,4,D}, {S,1,4,5,D}, {S,1,4,2,3,D}…*

### Design of Fitness Function

Genetic algorithms use the fitness value of each individual in a population to perform a search. The selection of the fitness function directly affects the convergence speed of the algorithm and whether the optimal solution can be found. Taking the highest network link stability, the maximum link bandwidth, and the most residual power of the nodes as the optimization parameters, and therefore the maximization problem, the fitness function is transformed from the objective function, and the objective function is converted into the fitness function, which can be obtained by Equation (4).

(4) $$\begin{array}{l} Fit\left(f\right)=Max(R_{l})=Max\sum_{\begin{array}{c} i\\ j\\ i\neq j \end{array}}\;\\ \left(\omega_{1}LS_{ij}+\omega_{2}\left(P_{i}+P_{j}\right)+\omega_{3}B_{ij}\right) \end{array}$$

### Select Operation

In order to avoid premature maturation of the algorithm and to ensure that better individuals can directly enter the next-generation population, a hybrid retransition-free selection operator is proposed here, which is a combination of two selection methods in a certain ratio of birth, followed by steady-state reproduction without retransition on this basis. The first way is tournament selection, and the proportion is α. The second way is roulette selection, and the probability of an individual being selected is proportional to its fitness value, and the proportion is 1 − α. After selection is completed, the first judgment is whether the individual is duplicated with the existing individuals in the population, and if it is, it is discarded.

### Crossover Operations

Crossover is the process of recombination and replacement of parts of the structure of two parent individuals to create a new individual. The recombination process can be between the chromosomes of the parent individuals or between the chromosomes for recombination of gene fragments. Because the chromosome genes in this paper are encoded with variable length codes, the traditional genetic algorithm of single point crossover is not applicable here. In network routing, crossover is used to select different paths between nodes, so crossover of gene fragments between chromosomes is adopted here. The crossover steps for the setup are as follows.

Select any two routes among all the routes to be selected and check all nodes in both routes. If there are nodes in both routes that pass through in common, then there is a common gene in both routes and the next crossover operation can be performed. If there is no common passing node in the two routes, then the two routes cannot perform the next crossover operation. If there are more than one common passing node in the two routes, the crossover operation starts from the first common passing node and ends at the last common passing node. If there is only one common passing node in the two routes, the common node is selected as the destination node.

Suppose for two chromosomes in generation *t*: *R*_*i*_*(t)* = *{S,1,3,D}, R*_*j*_*(t)* = *{S,1,2,3,4,D}*, nodes *1* and node *3* are the nodes that pass in common in the path and After the crossover operation, the next generation chromosome is: *R*_*i*_*(t* + *1)* = *{S,1,2,3,D}, R*_*j*_*(t* + *1)* = *{S,1,2,4,D}*.

### Mutation Operation

To address the problem of premature maturation caused by random variation in traditional genetic algorithms, a heuristic multiple variation operator is proposed here to explore the unknown region and suppress premature maturation, i.e., to seek the individual with the lowest value in its middle generation into the next generation by multiple heuristic variation. The mutation process is as follows.

Step 1: When mutation occurs, first check whether its intermediate nodes *i* and *I* + *2* are not in the routing range of the nodes.
Step 2: If yes, mutate the nodes that are not in the route range, and then return to step 1 until the route is a viable route.
Step 3: If not, randomly mutate its middle and record *M*_*i*_ into *R*.
Step 4: If *R* is not empty, select a random point from *R* to delete; otherwise, select a random point in the path to delete.
Step 5: Return to step 1 for multiple mutations (the number of mutations is generally taken as 3 or 4), and finally seek the individual with the lowest value in its middle generation to enter the next generation.

Assume that for generation t chromosome is *R(t)* = *{S,1,2,3,D}* and if node *(2,3)* is the mutation node, then the chromosome mutation in generation *t* + *1* is *R(t* + *1)* = *{S,2,3,D}*.

## Genetic-Algorithm-Based Routing

### GAR Route Searching

To initialize the route lookup process, the originating node *S* sends a ROUTE REQUEST (RREQ) as a single local broadcast packet, which is received by all nodes currently within the transmission coverage of node *S*. Each RREQ identifies the originating and destination nodes of the route lookup. Each RREQ identifies the originating node and the destination node for this route lookup. When another node receives this RREQ, it initiates the exchange of information on link stability, link bandwidth, and current energy of the node between neighboring nodes. At the same time, if this node is the destination node of this route lookup, then this node sends a ROUTE REPLY (RREP) back to the originating node of this route lookup, and after receiving the RREP, the originating node starts the GAR optimized route lookup procedure. If the node receiving the RREQ is not the destination node, then the node forwards the RREQ according to the local broadcast packet method and starts the neighbor node information exchange procedure.

The GAR route search steps are as follows:

Step 1: Initialize the chromosome based on the network node *ID*;
Step 2: Enter the next generation according to the individual adaptation value;
Step 3: Crossover operation according to the set crossover probability;
Step 4: Perform mutation operation according to the set mutation value;
Step 5: If the end condition of the algorithm is met, go to step 6, otherwise, go to step 2;
Step 6: Output the optimal chromosome as the satisfactory solution of the problem.

The pseudo code of genetic algorithm is shown in Table 1.

**Table 1:** GAR pseudocode.

BEGIN: *I* = 0; //Evolutionary population generation Init P(I); //Initializing the population Fitness P(I); //Fitness function While (i < =Genetic Generations) { I++;
Operation P(I); // Cross and mutation
Fitness P(I);
} // If the termination condition is not met, the search continues END.

### Route Maintenance

GAR still uses Acknowledgment to determine that a link is capable of data transmission. After a node receives an acknowledgment from an adjacent node, it can request that this adjacent node refrain from making an acknowledgment for a brief period of time, unless the network interface connecting a node to this adjacent node always receives an acknowledgment in response to a single destination stream. If the number of retransmissions of an acknowledgment request has reached the maximum allowed number of retransmissions and still no answer is received, the sending node considers that the link from which it reaches the next-hop destination node is currently broken and then removes this broken link from its routing memory, while sending back a ROUTE ERROR (RERR) to each such node. To reduce the burden on the node, instead of starting a GAR when looking for a route at the point of disruption, it will just find an available route for route repair.

## Simulation and Discuss

### Performance Parameters

To evaluate the performance of GAR, we perform comparative simulation to validate the three types of routing, GAR, AODV, and DSR, and focus on the following three important performance metrics for comparative evaluation (Quy et al., 2019).

(1) Control overhead: Control overhead is the ratio of routing messages (protocol packets) to the total communication data (protocol packets and data packets) in the network, i.e., the ratio of protocol packets sent and forwarded by all nodes to their data packets. This metric reflects the impact of routing protocols on network communication.
(2) Throughput: Throughput is the rate or number of packets of data transmitted throughout the network per unit of time. It is primarily the remaining bandwidth available to a network application between two nodes in the network at a given moment. That is, the maximum rate that a node can accept in the absence of frame loss.
(3) Average delay: Delay is the time it takes for a message or packet to travel from one end of a network to the other. It includes sending delay, propagation delay, processing delay, and queuing delay. For FANETs, propagation delay is the main concern.

### Simulation Parameters

The performance of GAR, AODV, DSR is compared and analyzed using NS3 simulation simulator. NS3 is an open source network simulator running on Linux that makes it easy to build networks that conform to the characteristics of FANETs (NS3 Network Simulator, 2011). The simulation is set up according to the hierarchical model as follows, all simulation parameters are shown in Table 2. In the simulation scenario built with Linux and NS3, each simulation time is 1200 s. Simulation scenario settings: nodes in the simulator are distributed according to a grid with 100 m sides; the communication radius of nodes is set to 500 m, the size of nodes is fixed to 10 × 10, i.e., the area where nodes move is limited to a rectangle of 2500 × 2500 m, and nodes are connected with a maximum of 20 The number of connections randomly generates data transmission requirements. The nodes use the random waypoint mobility model (Maan and Mazhar, 2011; Kumari et al., 2015; Bujari et al., 2017), which chooses a random direction for each node, moves at a set speed for a period of time and then randomly chooses another direction to continue moving, directly to the end of the simulation. The same random number seed is set to ensure that the nodes run the same trajectory in each simulation.

**Table 2:** Simulation parameters.

**Parameters**	**Value**
Simulator	Linux + NS3
Simulation times	1200 s
Nodes	10 × 10
Simulation area	2500 × 2500 m
Max of CBR Connections	20
MAC layer	802.11g FreeSpace model
Channel type	Wireless channel
Channel bandwidth	11 Mbps
Transmission speed	2 Mbps
Transmission range	500 m
Network layer	GAR, AODV, DSR
Transfer Models	WaveLan
Mobility model	Random waypoint mobility

### Simulation Results

**Figure 3:**
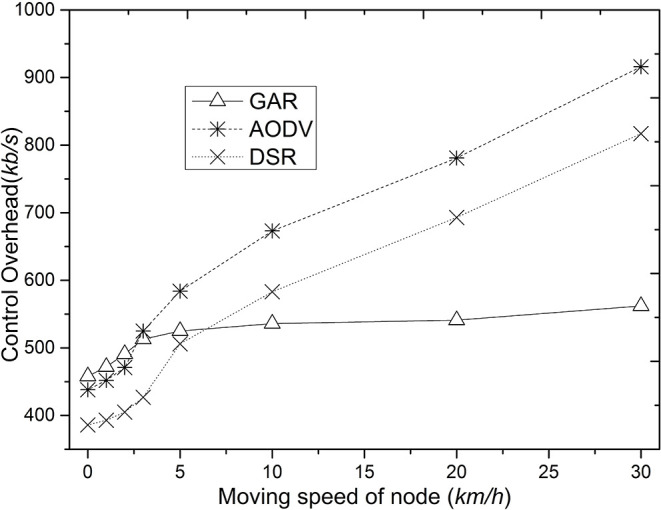
Comparison of control overhead as node speed increases.

Figure 3 shows the comparison of control overhead of GAR, AODV, and DSR. The control overhead of GAR is even higher than that of AODV and DSR in the case of low node movement speed, because when the node movement speed is low, the probability of routing disruption in the network is smaller, while GAR requires more message passing between nodes during the initial route finding, which results in more control messages in the network. However, as the node movement speed increases, AODV and DSR have a higher probability of communication link breakage than GAR because the stability of the link between nodes is not considered. In AODV and DSR, when the communication route breaks, the source node usually needs to restart the route finding in order to re-form a communication route, which causes more control overhead in the network.

**Figure 4:**
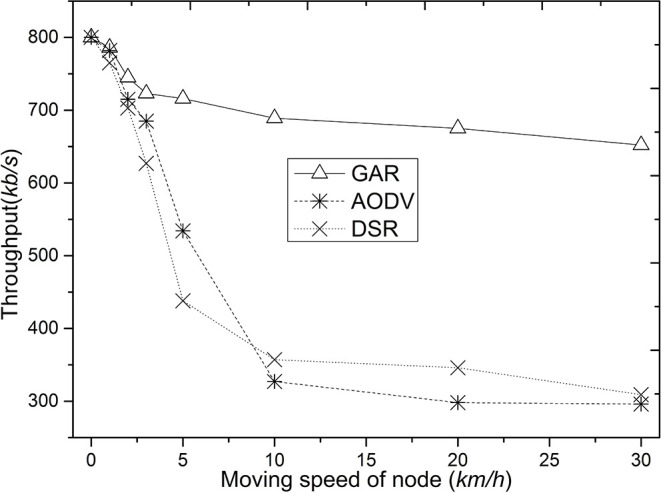
Comparison of throughput as node speed increases.

Figure 4 shows the data throughput comparison of GAR, AODV, and DSR. GAR shows better data throughput compared to AODV and DSR because GAR takes into account the stability of the route, energy of the node, etc., the route quality is higher, the movement of the node has less impact on the route, and therefore the route update control message occupies less of the channel. Whereas, in AODV and DSR, routes are selected based on minimum delay during route establishment. The route is not a stable route even though it is the best route at a certain point in time. In contrast, the interrelationship between the nodes on the route selected by GAR is more stable and its energy structure is more balanced. This routing method improves the lifetime of the selected route and avoids the bottleneck problem and packet collision problem at the intermediate nodes of the route, which ultimately improves the network data throughput.

**Figure 5:**
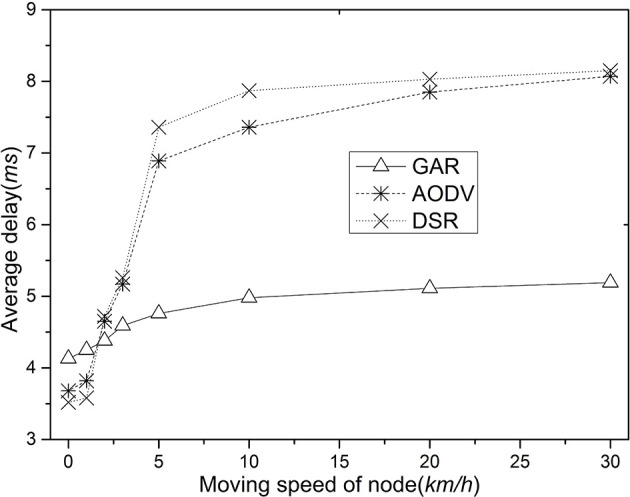
Comparison of average delay as node speed increases.

Figure 5 shows the average delay comparison for GAR, AODV, and DSR. As the node movement speed increases, the latency of the three routes increases, with AODV and DSR showing a more pronounced performance than GAR. The reason for this is that there are fewer packet collisions in the intermediate nodes of the network using GAR, and thus the packet delay is reduced. In AODV and DSR, after the intermediate node is disconnected, the packet must wait until a new route is established before it can be delivered, which naturally increases the packet transmission delay.

## Conclusion

Improving the lifetime and performance of the network from the perspective of improving routing has been the focus of research on FANETs. The stability of FANETs routing, the bandwidth of the link, and the energy of the nodes are fully considered therefore, the search of the route is performed using genetic algorithm on this basis to form GAR, and the simulation results show that GAR can effectively improve the stability of the route, which in turn improves the performance of the network and improves the lifetime and availability of the network. It is worth noting that we did not consider the energy consumption from the computation of the algorithm in a realistic environment, which will be the focus of our next research.

## Data Availability Statement

The original contributions presented in the study are included in the article/supplementary material, further inquiries can be directed to the corresponding authors.

## Author Contributions

This manuscript is mainly completed by XW, HY, WTH. XW designed an improved genetic algorithm and simulated it. HY and WTH analyzed the simulation results. XW wrote this manuscript, HY and WTH checked and reviewed the manuscript. All authors read and approved the final manuscript.

## Conflict of Interest

The authors declare that the research was conducted in the absence of any commercial or financial relationships that could be construed as a potential conflict of interest.
